# Periosteal new bone formation in Klippel-Trénaunay syndrome: a case report

**DOI:** 10.1186/s12887-020-02298-0

**Published:** 2020-08-19

**Authors:** Xiang Fang, Wenli Zhang, Zeping Yu, Fuguo Kuang, Bin Huang, Hong Duan

**Affiliations:** 1 Department of Orthopedics, West China Hospital, Sichuan University, 37 Guo Xue Lane, 610064 Chengdu, Sichuan People’s Republic of China; 2 Department of Orthopedics, People’s Fourth Hospital of Sichuan Province, Chengdu, Sichuan People’s Republic of China; 3 Department of Vascular Surgery, West China Hospital, Sichuan University, Chengdu, Sichuan People’s Republic of China

**Keywords:** Bone tumors, Children, Definite diagnosis, Periosteal reaction

## Abstract

**Background:**

Klippel-Trénaunay syndrome (KTS) is a complex congenital vascular disorder, typically accompanied by port-wine stains, varicose veins, and limb hypertrophy. This paper reports a rare and unusual clinical condition of periosteal reaction in a pediatric case of KTS. Although periosteal new bone formation is not rare in children, as is KTS, their dual occurrence or the presentation of the former due to KTS has not been previously documented. Our objective in this study is to highlight the potential association between periosteal new bone formation and KTS, as well as to help physicians consider this association when bone neoplasm has been ruled out.

**Case presentation:**

A 7-year old girl, initially presented with a persistent mild swelling in her left shank, with no abnormalities in the X-ray of the tibiofibular. However, after a few consults and examinations, 7 weeks later, a 17 cm-long periosteal new bone formation along the left tibia and diffused dilated vessels in the left shank were revealed by the radiological examination. Not knowing the true nature of the fast-growing lesion in a typical case of KTS was worrying. Therefore, a core needle biopsy was performed. The test demonstrated a possible parosteal hemangioma. Following further investigation through an excisional biopsy, and a pathological analysis, hyperplasia of the bone tissues with no tumor cells was revealed. Thereafter, an elastic stocking treatment was prescribed. During the first two-year follow-up, recurrence of the mass or sign of progression of KTS was not observed.

**Conclusions:**

Periosteal new bone formation is a potential manifestation of KTS. Based on the conclusive pathological results of the excisional biopsy, invasive examinations and surgeries could be avoided in future KTS-subperiosteal lesion manifestations.

## Background

Klippel-Trénaunay syndrome (KTS) in childhood is well-documented and commonly characterized by port-wine stains, varicose veins, and the overgrowth of long bones and soft tissues [1, 2]. In this report, we describe an atypical pediatric case of KTS, in which a 17 cm-long periosteal new bone formation of the tibia developed rapidly within 7 weeks. Periosteal new bone formation is very common in pediatric bone neoplasm, especially bone malignancy; however, it rarely occurs in cases of KTS. Therefore, this presentation has not been described in extant KTS-related literature. Our objective in this study is to highlight the potential association between periosteal new bone formation and KTS, as well as to help physicians consider this association when bone neoplasm has been ruled out.

## Case presentation

**Fig. 1:**
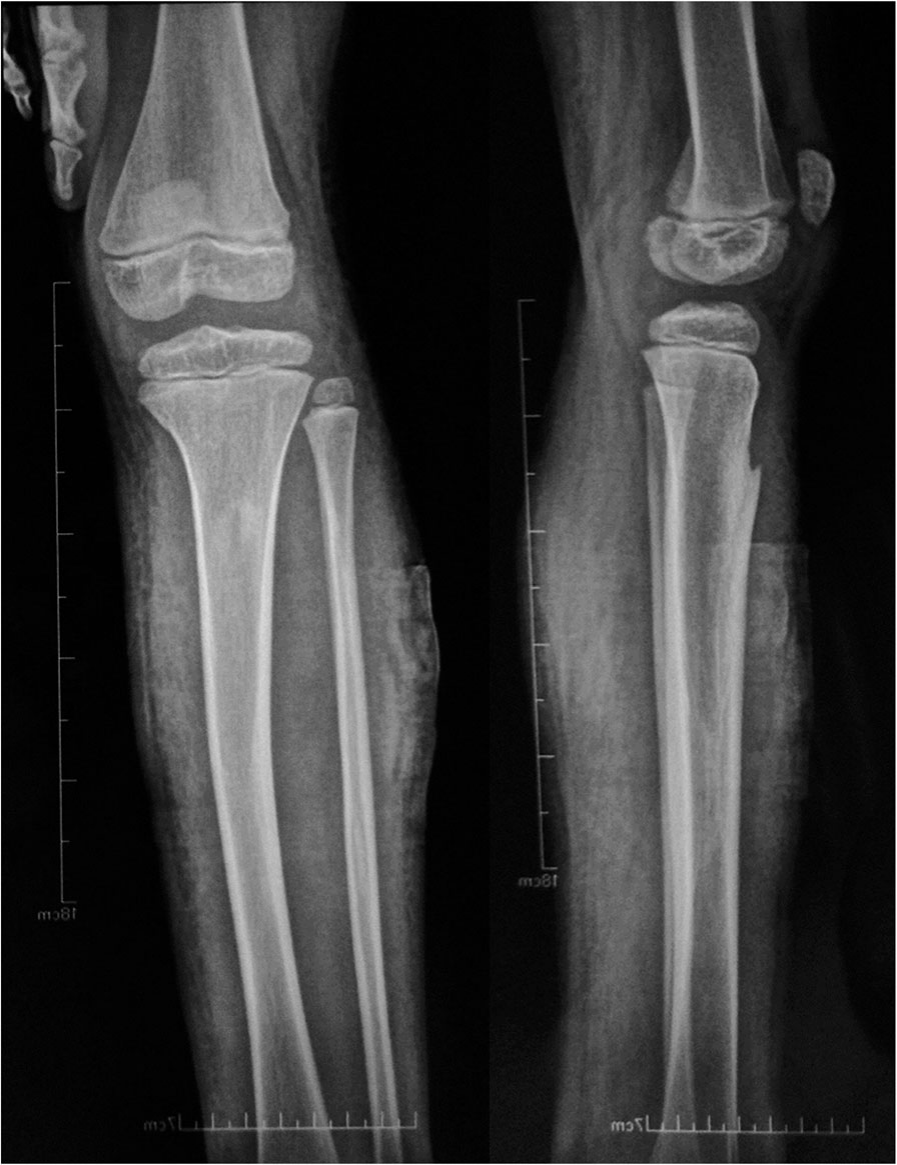
Initial radiograph of the tibiofibular shows no abnormalities

A 7-year old girl presented to the local hospital with mild swelling in her left shank that had persisted for one week. A radiograph of the tibiofibular showed no abnormalities (Fig. 1), and a herbal remedy for external use was applied. One week later, the shank swelling aggravated, and she presented to our hospital. On examination, apart from the swollen shank, we also observed multiple port-wine stains and limb overgrowth (longer extremity and larger foot). Ultrasonography revealed great/lesser saphenous vein thrombosis in the lower left leg and an ill-defined hypoechoic mass containing flaky hyperechoic foci near the left tibia. Based on the aforementioned findings, a clinical diagnosis of KTS was established. The use of low-molecular weight heparin and mucopolysaccharide polysulfate cream was initiated.

**Fig. 2:**
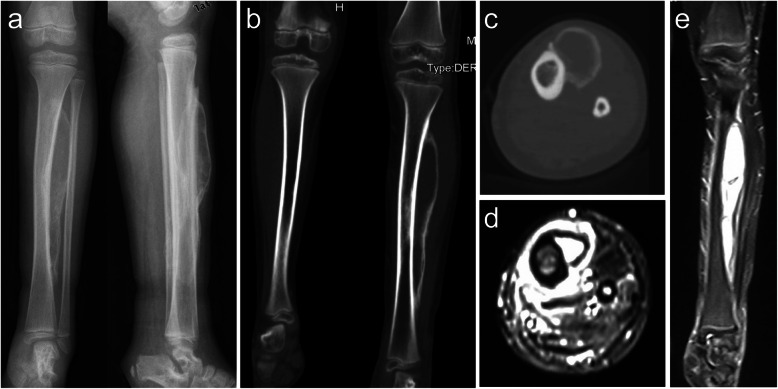
Radiographs and computed tomography images of the tibiofibular (obtained 7 weeks after the initial radiograph) reveal a parosteal high-density lesion with well-defined borders along the long axis of the left tibia (**a, b, c**). T2-weighted magnetic resonance imaging scans of the lower legs show heterogeneous high-signal intensity in the anterolateral aspect of the tibia, with diffused dilated vessels in the left shank (** d, e**)

The patient re-admitted 6 weeks later, reporting a palpable hard mass in the anterolateral left shank and complete resolution of the swelling. Radiological examination revealed a 17-cm periosteal new bone formation along the left tibia and multiple dilated vascular structures in the left shank (Fig. 2). Coagulation status was normal. No fever, allergies, severe pain, or a recent history of trauma were presented.

Bone neoplasm was first suspected but subsequently ruled out due to the regular pattern of periosteal new bone formation without soft tissue mass, bone destruction, and symptoms. However, concerning the fast-growing lesion in KTS, we performed a core needle biopsy, which revealed a possible parosteal hemangioma. Consequently, an excisional biopsy of the lesion was performed and intraoperatively, only regularly thickened eggshell-like hard tissues and blood clots in the cavity of the lesion were found, Pathological analysis revealed hyperplasia of the bone tissues with cystic wall-like structures, old hemorrhage with hemosiderin deposition, and no tumor cells. Thereafter, an elastic stocking treatment was prescribed. During the first two-year follow-up, recurrence of the mass or sign of progression of KTS was not observed.

## Discussion and conclusions

**Fig. 3:**
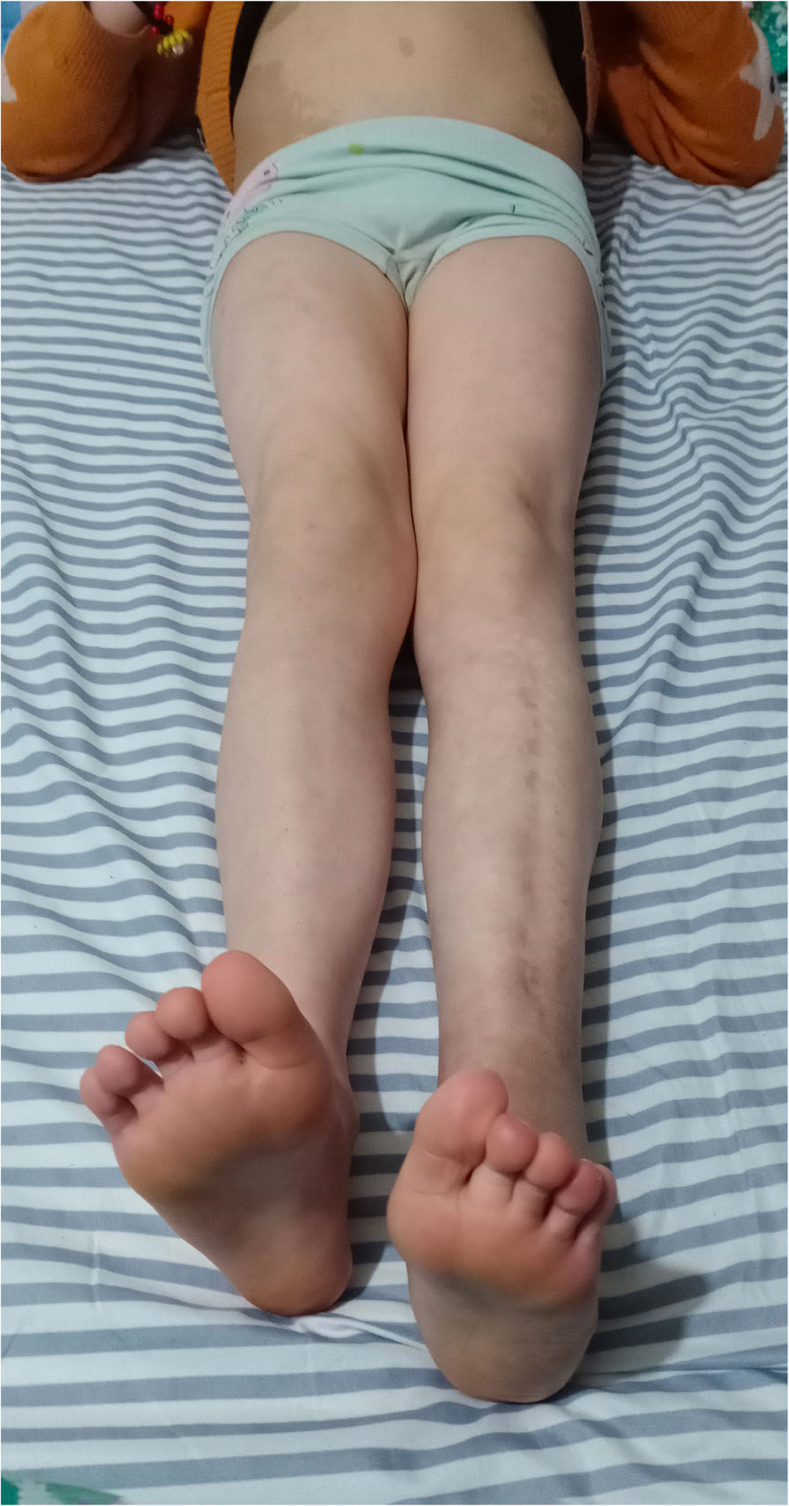
Photograph of the patient (taken 2 years postoperatively) shows multiple port-wine stains and limb overgrowth

First described by Maurice Klippel and Paul Trénaunay in the year 1900, KTS is estimated to affect approximately one in 30 000–100 000 liveborn neonates [3–5]. It is a complex congenital vascular disorder accompanied by capillary malformation (port-wine stains), venous malformation (varicose veins), and overgrowth of the long bones and soft tissues, usually involving a single lower extremity [1]. Our patient was born with port-wine stains from the abdomen to toes and minor limb discrepancy that became more apparent over time (Fig. 3).

The actual pathogenesis of KTS is not completely understood. A somatic mosaic mutation in phosphatidylinositol-4,5-bisphosphate 3-kinase catalytic subunit alpha (PIK3CA) is hypothetically a major potential cause of KTS [6]. However, other factors, including the mutations in the angiogenic factor with G patch and FHA domains 1 (*AGGF1*), Ras p21 protein activator 1 (*RASA1)*, and Krev interaction trapped 1 (*KRIT1*) genes, and alterations in the fetal mesoderm for intrauterine damage, may also be involved in disease pathogenesis [6–12]. However, our patient denied any intrauterine damage and her parents did not allow her to undergo gene mutation tests.

Typical KTS cases are diagnosed based on physical examination findings without imaging, laboratory, or genetic testing [13]. However, it should be differentiated from Parkes–Weber syndrome—a high-shunt fast-flow arteriovenous malformation—by checking for the presence of a significant arteriovenous fistula. In our patient, the diagnosis was made based on her inborn port-wine stains, limb overgrowth, vasodilation in her right lower limb, as detected on MRI, and absence of an arteriovenous fistula in ultrasonography.

There is no cure for KTS; therefore, treatment is symptomatic. Nonoperative modalities play a major role in most symptomatic KTS patients. Compression and elevation are the fundamental bases for lower extremity chronic venous disease. Although patients with KTS are at an increased risk of thromboembolic events, anticoagulation therapy is not indicated in the early clinical course. However, it should be initiated following the presentation of deep venous thrombosis, like at the time of the first hospital presentation of our patient, or for prophylaxis during the perioperative course [14]. The absolute indications for operative vascular intervention are persistent hemorrhage, acute thromboembolism, and refractory ulcerations, while the relative indications are pain, cosmetic, limb asymmetry, swelling secondary to venous insufficiency, and functional impairment [15]. When limb length discrepancy is < 1.5 cm, heel inserts or compensatory shoes can be used to improve the limp and to avoid possible scoliosis. However, when the discrepancy is > 2 cm, orthopedic osteotomy or epiphysiodesis should be considered [16]. Our patient had a mild form of KTS. She was mostly asymptomatic, even prior to the use of the elastic stocking. Despite the discrepancy in the limb length, she could walk and run normally, without limping, with heel inserts. However, as her limb overgrowth gradually became more apparent, she was constantly annoyed by her larger right foot when buying new shoes.

Atypical clinical manifestations of KTS reportedly include hypersplenism, nephrotic syndrome, cerebral cavernous angioma, and puerperal hemorrhage [17–19]. Bone involvement in KTS is commonly noted and typically manifests as circumferential hypertrophy, longer extremities, ectrodactyly, polydactyly, syndactyly, camptodactyly, and clinodactyly, and in rare cases, intraosseous vascular malformation [1, 2, 20, 21]. However, to the best of our knowledge, this report presents the first case of KTS with periosteal new bone formation.

Periosteal new bone formation, also called periosteal reaction, is a nonspecific response of the periosteum to underlying “irritation,” which typically presents not only in patients with benign and malignant tumors, as well as osteomyelitis and thalassemia. A nonaggressive periosteal bone formation is usually slow-growing with thin, solid, thick and irregular, or septated imaging features, while fast-growing masses with laminated (onion skin), spiculated (perpendicular/hair-on-end and sunburst), disorganized, and Codman triangle images are generally found in the aggressive periosteal reactions [22]. Our patient developed a rapidly enlarging osseous mass with nonaggressive periosteal new bone formation, thus not implying malignancy. However, the true nature of the lesion remained unknown before final excision, as there are reports on tumors detected in patients with KTS, including malignant peripheral nerve sheath tumors, angiosarcomas, astrocytomas, hemangiopericytomas, hemangiomas, and meningiomas [23–28]. Moreover, isolated hemihypertrophy, a major clinical manifestation of KTS, is a potential risk factor for developing neoplasms, although the risk of embryonal cancer is reportedly not higher in children with KTS [29–31].

The precise pathophysiology for the large periosteal new bone formation in KTS remains unknown to date. However, due to the KTS-related massive vascular malformation, we speculated that the spontaneous rupture or minor trauma of the diseased capillaries on the periosteum led to subperiosteal bleeding, which further lifted the periosteum. The new bone was suspected to generate by the periosteum; accordingly, bone tumor was suspected. Although a serious malignancy was not found in radiology, the fast-growing lesion in a typical KTS case still worried physicians. However, with definitive pathological results, invasive examinations and surgical interventions may be avoided for KTS patients with subperiosteal lesions in the future.

## Data Availability

All data generated or analysed during this study are included in this published article

## Abbreviations

KTS: Klippel-Trénaunay syndrome
PIK3CA: Phosphatidylinositol-4:5-bisphosphate 3-kinasecatalytic subunit alpha
AGGF1: Angiogenic factor with G patch and FHA domains 1
RASA1: Ras p21 protein activator 1
KRIT1: Krev interaction trapped 1
